# Novel leptin OB3 peptide-induced signaling and progression in thyroid cancers: Comparison with leptin

**DOI:** 10.18632/oncotarget.8505

**Published:** 2016-03-30

**Authors:** Yu-Chen SH Yang, Yu-Tang Chin, Meng-Ti Hsieh, Hsuan-Yu Lai, Chien-Chih Ke, Dana R. Crawford, Oscar K. Lee, Earl Fu, Shaker A. Mousa, Patricia Grasso, Leroy F. Liu, Heng-Yu Chang, Heng-Yuan Tang, Hung-Yun Lin, Paul J. Davis

**Affiliations:** ^1^ Joint Biobank, Office of Human Research, Taipei Medical University, Taipei, Taiwan; ^2^ Taipei Cancer Center, Taipei Medical University, Taipei, Taiwan; ^3^ PhD Program for Cancer Biology and Drug Discovery, College of Medical Science and Technology, Taipei Medical University, Taipei, Taiwan; ^4^ Biomedical Imaging Research Center, National Yang-Ming University, Taipei, Taiwan; ^5^ Center for Immunology and Microbial Disease, Albany Medical College, Albany, New York, USA; ^6^ Stem Cell Research Center, National Yang-Ming University, Taipei, Taiwan; ^7^ Department of Periodontology, School of Dentistry, National Defense Medical College, Taipei, Taiwan; ^8^ Pharmaceutical Research Institute, Albany College of Pharmacy and Health Sciences, Albany, New York, USA; ^9^ Department of Medicine, Albany Medical College, Albany, New York, USA; ^10^ Department of Biochemistry and Molecular Cell Biology, College of Medicine, Taipei Medical University, Taipei, Taiwan; ^11^ Graduate Institute of Medical Sciences, College of Medicine, Taipei Medical University, Taipei, Taiwan

**Keywords:** obesity, leptin, OB3-leptin peptide, cancer cell invasion

## Abstract

Obesity results in increased secretion of cytokines from adipose tissue and is a risk factor for various cancers. Leptin is largely produced by adipose tissue and cancer cells. It induces cell proliferation and may serve to induce various cancers. OB3-leptin peptide (OB3) is a new class of functional leptin peptide. However, its mitogenic effect has not been determined. In the present study, because of a close link between leptin and the hypothalamic-pituitary-thyroid axis, OB3 was compared with leptin in different thyroid cancer cells for gene expression, proliferation and invasion. Neither agent stimulated cell proliferation. Leptin stimulated cell invasion, but reduced adhesion in anaplastic thyroid cancer cells. Activated ERK1/2 and STAT3 contributed to leptin-induced invasion. In contrast, OB3 did not affect expression of genes involved in proliferation and invasion. *In vivo* studies in the mouse showed that leptin, but not OB3, significantly increased circulating levels of thyrotropin (TSH), a growth factor for thyroid cancer. In summary, OB3 is a derivative of leptin that importantly lacks the mitogenic effects of leptin on thyroid cancer cells.

## INTRODUCTION

High-fat diets in the obese may cause multiple molecular factors to act synergistically to increase the risk of colon cancer associated with obesity [1]. Other cancers have also been shown to occur with increased frequency in obesity [2]. The obesity-related hormone, leptin, is secreted by adipose tissue and has been implicated in the onset and progression of several types of cancer, including colorectal cancer and cancers of the breast, endometrium, and esophagus [3]. Leptin is mitogenic for hematopoietic cells, normal and transformed epithelial cells and vascular endothelial cells [4, 5] and has been shown experimentally to stimulate proliferation of breast cancer, hepatoma and prostate cancer cells [6–9]. A component of safer diets in the future will be inclusion of factors that reduce the contribution of ciculating endogenous leptin to tumor biology. Recently, a new class of functional leptin peptides has been synthesized [10]. The effects of OB3 leptin-related synthetic peptides on energy balance and glucose homeostasis in ob/ob and db/db mice have been confirmed [11–13]. OB3 does not stimulate proliferation of human cervical cancer HeLa cells (Lin HY: unpublished observation), but additional information is needed about responses or lack of response of other cancer cells to OB3.

Leptin has important effects on the hypothalamic-pituitary-thyroid axis [14]. It stimulates the expression of thyrotropin-releasing hormone (TRH) in the hypothalamus and consequent production of thyrotropin (TSH). Leptin receptor (OBR) exists in all thyroid cancer cells. It is overexpressed in papillary thyroid cancer and is associated with tumor aggressiveness [15] and a higher incidence of lymph node metastasis [16]. Leptin acting via leptin receptor may regulate cell migration of thyroid cancer cells [15].

Leptin promoted thyroid cancer progression is associated with the Janus kinase 2 (JAK)-signaling transducer and activator of transcription (STAT)-3 signaling pathway and with STAT3 target gene expression [17]. Recruitment via the Src homology 2 domain to receptor phosphotyrosine peptide motifs facilitates STAT phosphorylation on a key tyrosyl residue by growth factor receptors and by JAK [18]. The phosphorylation induces STAT-STAT dimerization and nuclear translocation of the dimer, with eventual binding to specific DNA-response elements in the promoters of target genes and gene transcription [19]. The STAT3 signaling pathway is involved in thyroid cancer aggravated by obesity-related metabolic changes [17] and may be a potential therapeutic target in thyroid cancer. OB3 peptide also induces STAT3 phosphorylation by ERK1/2 and PI3K-dependent signal transduction [10].

Focal adhesion kinase (FAK) is a cytoplasmic tyrosine kinase that has crucial roles in integrin-mediated signal transduction and also participates in signaling from other cell surface receptors. Integrin signaling through FAK has been shown to promote cell migration in numerous studies. Increased levels of FAK expression have been correlated with the invasive and metastatic potential of several human tumors [20, 21]. Integrins have important functions in tumor angiogenesis and are major upstream activators of FAK. Blockade of integrin αvβ3 with monoclonal antibodies or small molecules significantly has been shown to reduce tumor angiogenesis in a variety of animal models [22, 23]. Integrin clustering activates FAK and leads to autophosphorylation at Tyr397, which is a binding site for src family kinases and PI3K [24]. In addition, leptin stimulates migration of colon carcinoma cells. This has been found to be accompanied by an increase of total FAK expression and a significant up-regulation of FAK phosphorylation at Tyr397, as well as Tyr576/577 [25].

Leptin may play a role in the development of thyroid cancers [16, 26, 27]. Whether it has prognostic value is undetermined and the molecular basis of its effects on thyroid cancer cells is also unclear. In the present study, we compared the signal transduction and other known mechanisms of action of leptin peptide with OB3 peptide actions in thyroid cancer cells. We found that leptin, but not OB3, stimulated the invasiveness of anaplastic thyroid cancer cells and reduced adhesion of papillary thyroid cancer cells. OB3 was found only to increase adhesion in papillary thyroid cancer cells. Thyrotropin (TSH) is a pituitary hormone that supports differentiated thyroid cancer [14, 28]; leptin, but not OB3, was found in the present studies to increase circulating TSH levels in intact mice.

## RESULTS

### OB3 peptide does not stimulate cell proliferation in thyroid cancer cell lines

**Figure 1:**
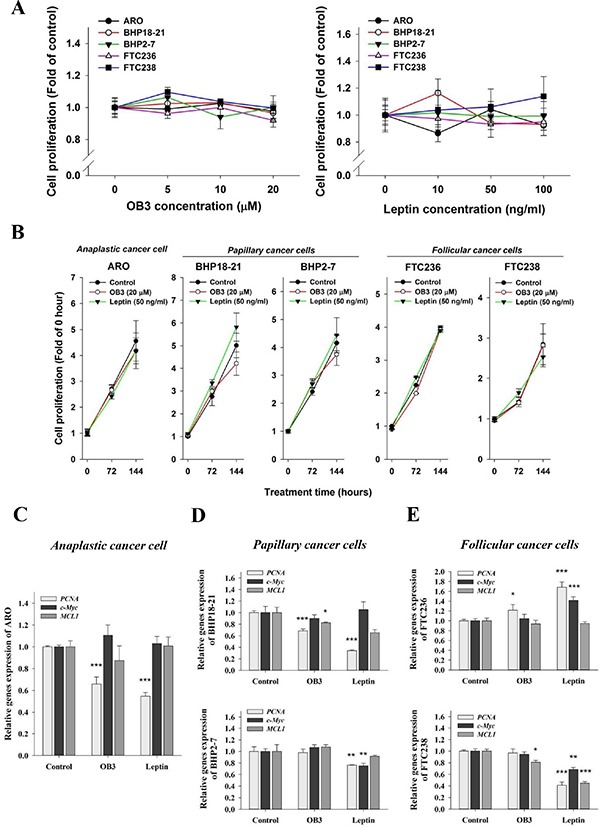
Effect of OB3 and leptin peptide on cell proliferation and proliferative gene expression in thyroid cancer cell lines (**A**) Cells were seeded in 96-well plates and treated with different concentrations of OB3 peptide (left) or leptin peptide (right), and with re-freshed medium daily for 144 h. Cell proliferation was examined by MTT assay. (**B**) Cells were seeded in 96-well plates and treated with leptin peptide or OB3 with re-freshed medium daily for 144 h. Cell proliferation was examined on 72 h and 144 h by MTT assay. (**C**–**E**). Aliquots of cells of different thyroid cancer cell lines were treated with either leptin or OB3 for 24 h. Cells were harvested and total RNA was extracted. qPCR was conducted for *PCNA*, *c-Myc* and *MCL1*, as described in the Materials and Methods. (*p < 0.05, **p < 0.01, ***p < 0.001, were compared with control).

Effect of OB3 peptide on cell proliferation in thyroid cancer cells was examined. Anaplastic, papillary and follicular thyroid cancer cells were treated with different concentrations of OB3 for 144 hours with refreshed medium and OB3 peptide added daily. The results from MTT assay showed that OB3 treatment did not induce significant changes in cell growth of any of the thyroid cancer cell lines examined (Figure 1A, left).

Similar studies were conducted with leptin to compare proliferative effects of OB3 and leptin in thyroid cancer cells. Thyroid cancer cells were treated with different concentrations of leptin for 144 hours with re-freshed leptin daily. Results presented in Figure 1A (right) indicated that leptin did not induce cell proliferation in thyroid cancer cells. These results were contrary to previous reports in which leptin peptide has proliferative effects [29, 30]. We next examined expression of proliferative genes induced by OB3 and leptin in thyroid cancer cells.

The time course effect of leptin or OB3 on cell proliferation was also investigated. Different thyroid cancer cells were treated with 20 μM OB3 or 50 ng/mL leptin for 144 hours with media and reagents refreshed daily. MTT assays were conducted at 0, 72 and 144 hours after treatment of OB3 or leptin. Results indicated that there was no significant change among proliferation in control, OB3- or leptin-treated cancer cells in 5 different types of thyroid cancer cells (Figure 1B).

To investigate the effect of leptin and OB3 on the gene expression involved in cell proliferation, we exposed the cells to leptin or OB3. Both OB3 and leptin reduced *PCNA* expression significantly and increased the expression of *c-Myc* and *MCL-1* slightly in anaplastic thyroid cancer cells (Figure 1C). In papillary thyroid cancer cell lines, OB3 and leptin reduced the expression of *PCNA* and *MCL1* in BHP18-21 (Figure 1D), however, only leptin reduced the expression of *PCNA* and *c-Myc* in BHP2-7 cells (Figure 1D). In follicular thyroid cancer cells, leptin had more dramatic effects in gene expression than those of OB3; for example leptin increased the expression of *PCNA* and *c-Myc* in FTC236 cells but decreased the expression of *PCNA*, *c-Myc* and *MCL-1* in FTC238 cells (Figure 1E).

### Leptin and OB3 change the expression of genes involved in carbohydrate metabolism in thyroid cancer cells

**Figure 2:**
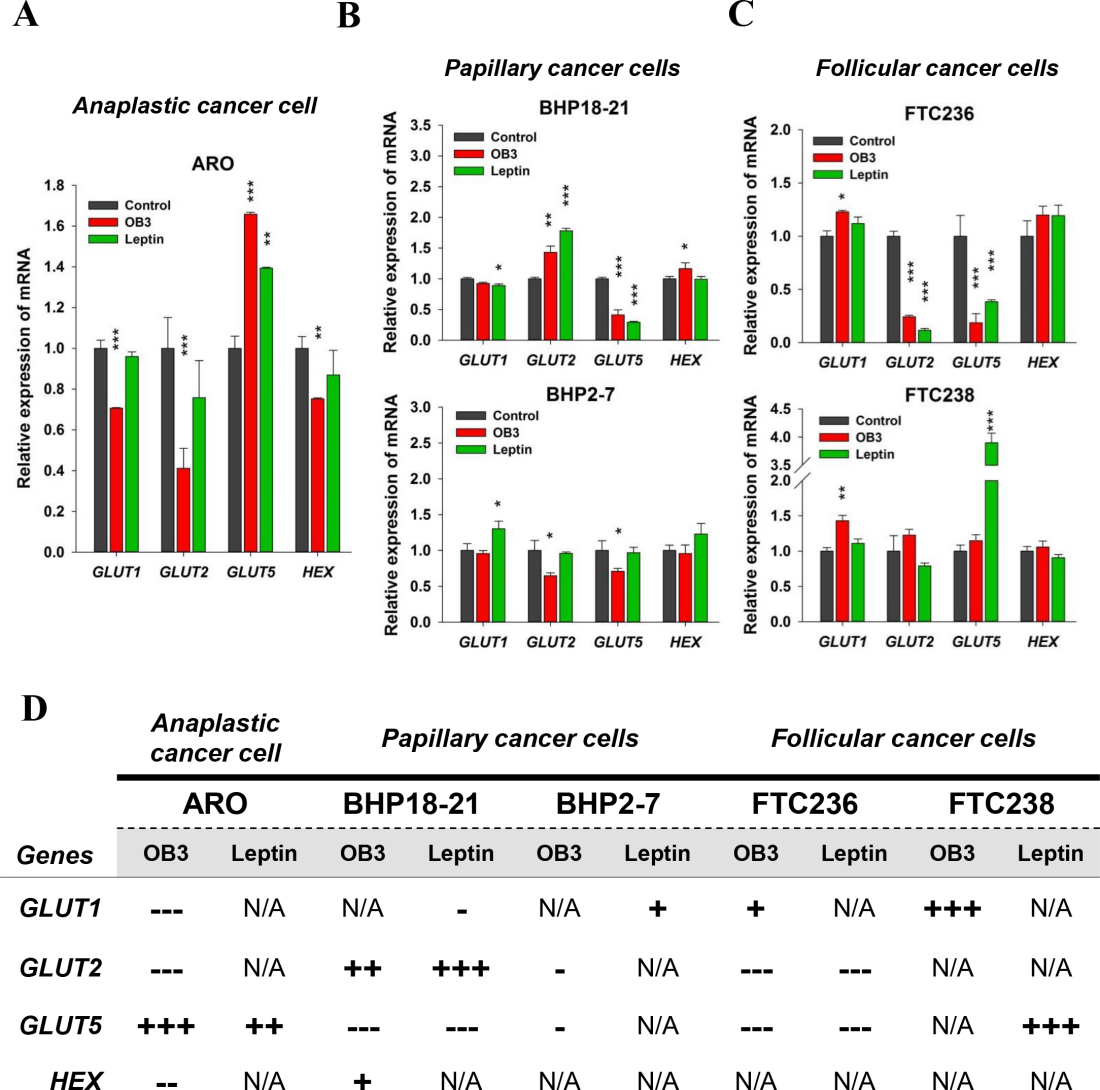
Effect of OB3 and leptin peptides on expression of genes relevant to metabolism in thyroid cancer cell lines (**A**) Anaplastic, (**B**) papillary, and (**C**) follicular thyroid cancer cells were treated with either leptin or OB3 for 24 h. Cells were harvested and total RNA was extracted. qPCR for *GLUT1*, *GLUT2*, *GLUT5*, and *HEX* was conducted as described in the Materials and Methods. (**D**) According to statistical analysis of qPCR, the comparison of OB3 and leptin on metabolism genes in thyroid cancer cell lines was shown. (*p < 0.05, **p < 0.01, ***p < 0.001, were compared with control).

Leptin affects the expression of genes relevant to carbohydrate metabolism [31]. In order to determine whether leptin and OB3 affect glucose metabolism-related gene expression in human thyroid cancer cells, we measured expression of glucose transporter (*GLUT1*, *GLUT2*, *GLUT5*) genes and the hexokinase 1 gene in a panel of cell lines. Transcription of *GLUT1*, *GLUT2* and hexokinase 1 (*HEX*) in anaplastic thyoid carcinoma (ARO) cells was inhibited by OB3 treatment (Figure 2A), whereas OB3 induced expression of *GLUT5* in these cells. Leptin induced *GLUT5* expression, but did not affect the remainder of the other genes examined (Figure 2A). In papillary thyroid cancer (BHP18-21) cells, OB3 significantly inhibited *GLUT5* transcription, but enhanced *GLUT2* and *HEX* expression. In the same cell line, however, treatment with leptin increased *GLUT2* expression, but significantly inhibited the expression of *GLUT1* and *GLUT5* (Figure 2B, upper panel). In anoher papillary thyroid cancer (BHP2-7) cell line, there was an inhibitory effect of OB3 on the expression of *GLUT2* and *GLUT5*, but leptin induced *GLUT1* transcription (Figure 2B, lower panel). In follicular thyroid cancer (FTC236) cells, both OB3 and leptin significantly reduced the expression of *GLUT2* and *GLUT5*, but only OB3 increased *GLUT1* expression (Figure 2C, upper panel). OB3 and leptin significantly induced the expression of *GLUT1* and *GLUT5*, respectively in FTC238 cells (Figure 2C, lower panel). The statistical analysis of qPCR results that compare actions of OB3 and leptin on metabolism-relevant genes in thyroid cancer cell lines is shown in Figure 2D. Thus, OB3 and leptin alter the expression of glucose metabolism-related genes in various types of thyroid cancer cells, but the actions of OB3 and leptin and patterns of genes affected are cell line-specific and may not related to cancer cell proliferation or invasiveness.

### Leptin, but not OB3, induces invasion in anaplastic thyroid cancer cells

**Figure 3:**
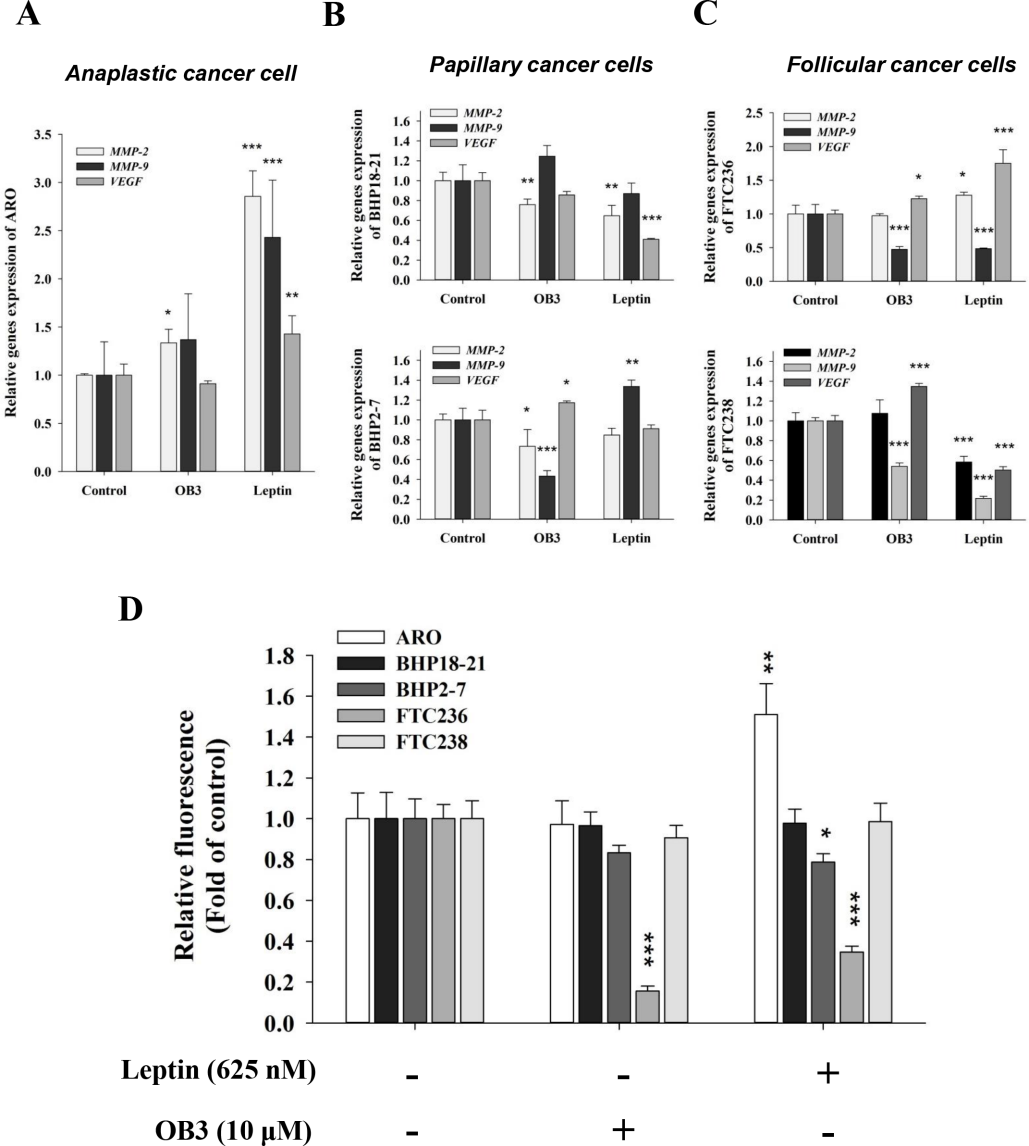
Effect of OB3 and leptin peptides on expression of genes relevant to invasion and cell invasion in thyroid cancer cell lines (**A**) Anaplastic (**B**), papillary and (**C**) follicular thyroid cancer cells were treated with either leptin or OB3 for 24 h. Cells were harvested and total RNA was extracted. qPCR for *MMP2*, *MMP9* and *VEGF* was conducted as described in the Materials and Methods. (**D**) Thyroid cancer cells (1 × 10^5^/well) were starved in 0.1% serum-containing medium with 0.625 μM leptin or 10 μM OB3 at 37°C for 4 h and seeded onto the upper chamber of the transwell using the Millipore system for cell migration. After 6 h, the cells were subjected to chemoattraction and migrated to the lower chamber. Migration was quantified using the fluorimetric detection system (Millipore). (Data were expressed as mean ± S.D. in triplicate. *p < 0.05, **p < 0.01, ***p < 0.001, were compared with control).

In anaplastic thyroid cancer cells, leptin induced the expression of *MMP2*, and *MMP9* which are involved in the invasion of cancer cells (Figure 3A). OB3 induced only *MMP2* significantly and *MMP9* marginally in anaplastic thyroid cancer cells (Figure 3A). However, the expression of *VEGF*, a principal contributor to angiogenesis, was enhanced by leptin but not OB3 in anaplastic thyroid cancer cells (Figure 3A). On the other hand, papillary (Figure 3B) and follicular (Figure 3C) thyroid cancer cells exhibited no significant increase in the expression of genes involved in the invasion in response to OB3 and leptin.

To verify these observations, we analyzed the effect of both leptin and OB3 on the invasive potential using Transwell assay. As shown in Figure 3D, the ability of anaplastic thyroid cancer cells to invade was remarkably increased when they were treated with leptin. In contrast, both papillary and follicular thyroid cancer cells treated with leptin did not increase invasiveness, compared to the untreated control. Interestingly, both OB3 and leptin inhibited the invasion in follicular thyroid cancer FTC236 cells (Figure 3D).

### Leptin induces the activation of signal transduction pathway in anaplastic thyroid cancer cells

**Figure 4:**
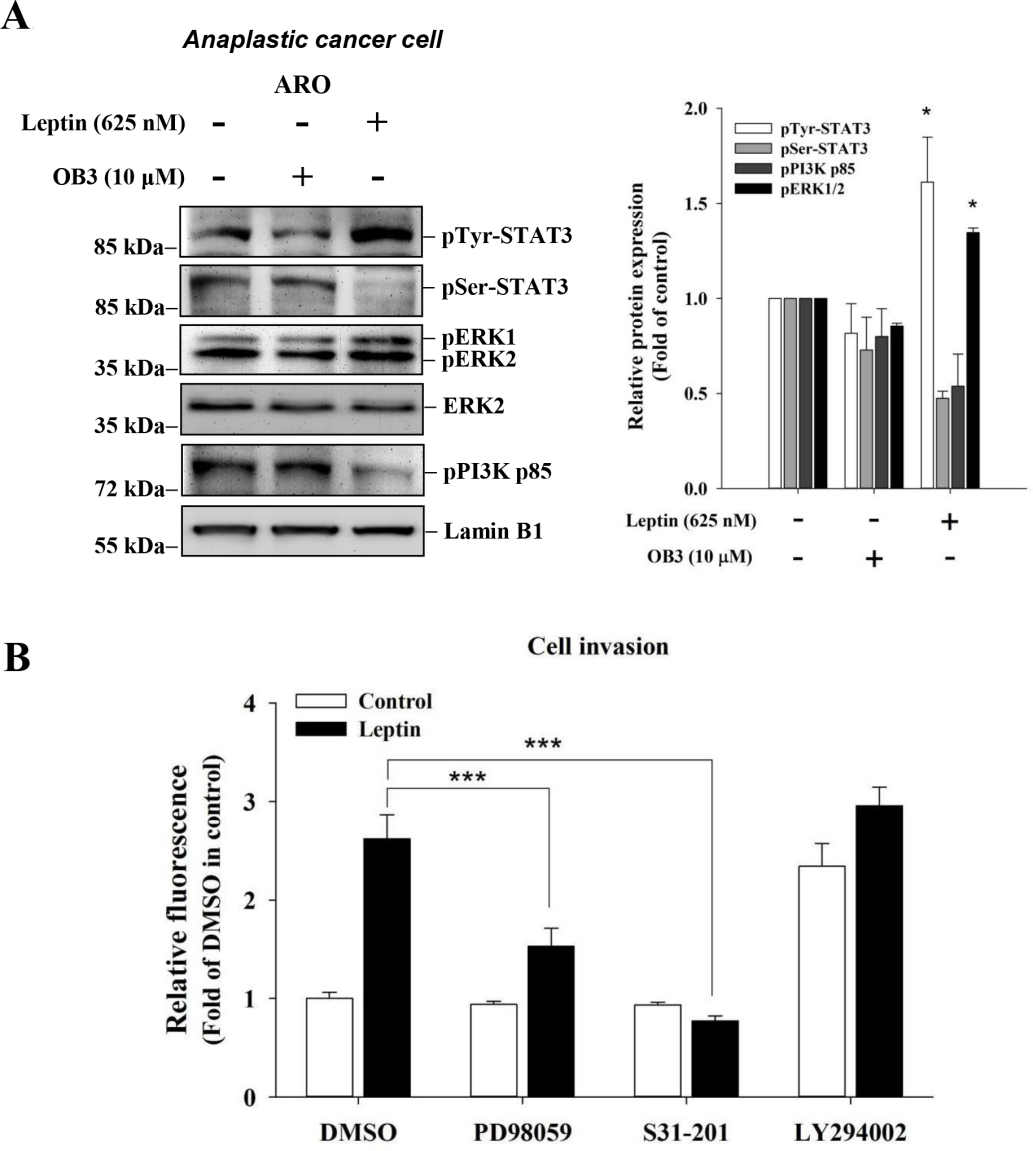
Signal transduction system activation is involved in leptin-induced invasion in anaplastic thyroid cancer cells (**A**) Anaplastic thyroid cancer cells were incubated with 0.1 or 10 μM OB3, or leptin for 30 min. Nuclear extracts were prepared, proteins separated by PAGE, then immunoblotted with anti-phosphorylated MAPK (pERK1/2), anti-phospho-PI-3K p85, pSer-STAT3 antibody, and pTyr-STAT3 antibody. β-Actin was used as an internal loading control. Immunoblots were visualized by enhanced chemiluminescence (Amersham Life Science, Arlington Heights, IL, USA) and digital imaging. Results represent the mean ± SEM of three independent experiments. *p < 0.05 compared to untreated control. (**B**) Anaplastic thyroid cancer cells (1 × 10^5^/well) were starved in 0.1% serum-containing medium with 0.625 μM leptin with or without PD98059 (10 μM), LY294002 or STAT3 inhibitor for one hr, and then incubated in the absence or presence of 10 μM leptin at 37°C for 4 h and seeded into the upper chamber of the transwell using the Millipore system for cell migration, respectively. After 6 h, the cells were subjected to chemoattraction and migrated into the lower chamber. A fluorimetric detection system (Millipore) quantitated movement. Data were expressed as mean ± S.D. in triplicate. (***p < 0.001, were compared with DMSO).

Leptin is known to activate PI3K [32]. We have shown that OB3 induces ERK1/2 and PI3K activation in HeLa cells. OB3 also induces phosphoryIation of Ser-727 STAT3 and Tyr-705 of STAT3 [10]. In order to determine if leptin/OB3 activated signal transduction in anaplastic thyroid cancer cells, cells were treated with OB3 (10 μM) or leptin (0.625 μM) for 4 h. Results shown in Figure 4A indicate that both OB3 and leptin activated ERK1/2 activation, but reduced PI3K phosphorylation (Figure 4A). In addition, phosphorylation of Tyr-705 in STAT3, but not Ser-727, was induced by leptin and OB3 in anaplastic thyroid cancer cells (Figure 4A). On the other hand, neither OB3 nor leptin activated the ERK1/2 or PI3K signal transduction pathways in follicular and papillary thyroid cancer cells (results not shown). When anaplastic thyroid cancer cells were treated with leptin in the presence or absence of specific signal transduction pathway inhibitors PD98059, LY294002 and an inhibitor of STAT3, S31-201, the leptin-induced cell invasion was inhibited by PD and S31-201, but not LY294002 (Figure 4B). These results indicated that leptin-induced phosphorylation of ERK1/2 and Tyr-705 of STST3 plays an important role in leptin-induced invasion of anaplastic thyroid cancer cells.

### Inactivated FAK is involved in leptin-induced anti-adhesion in anaplastic thyroid cancer cells

**Figure 5:**
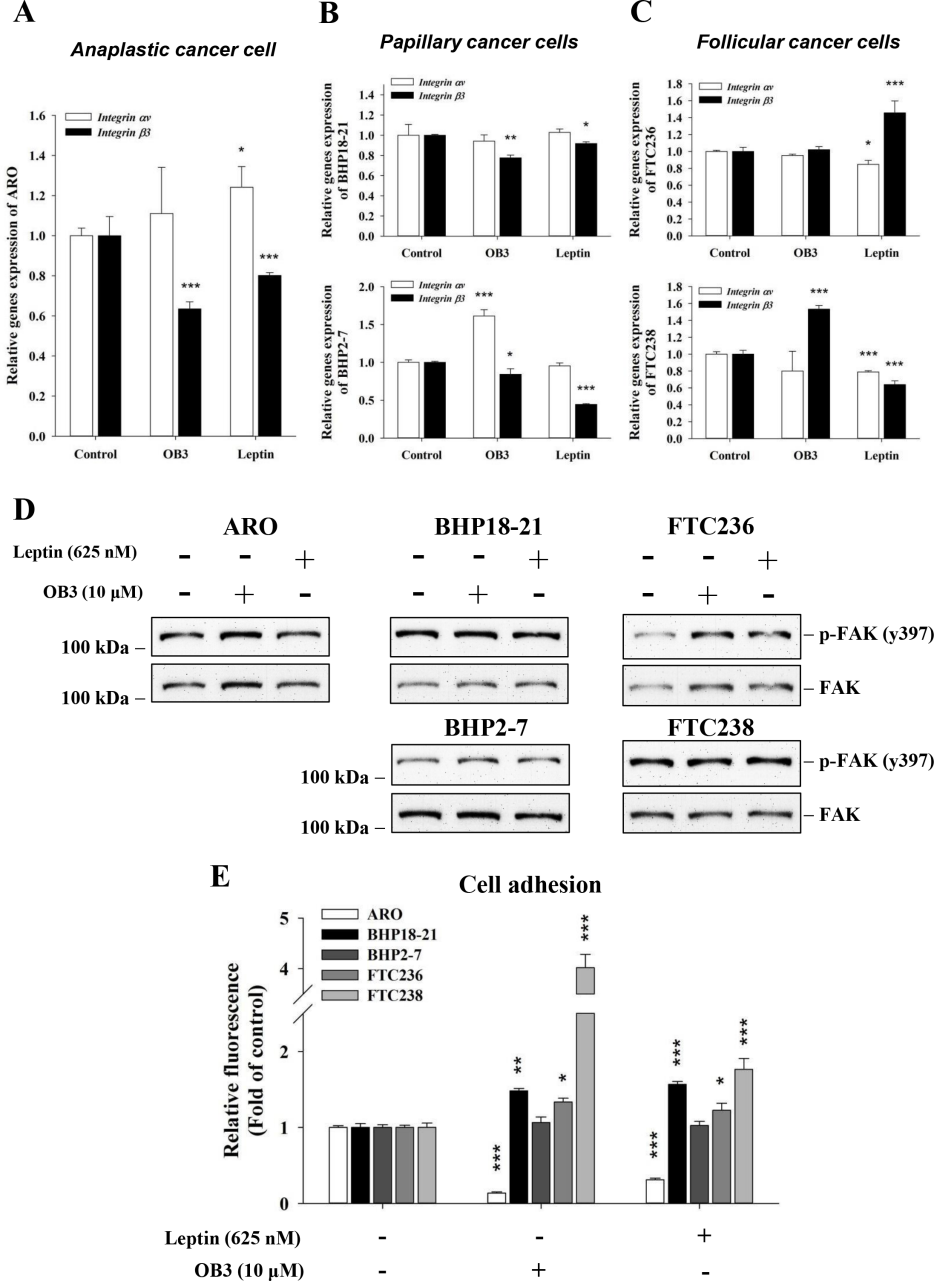
Effect of OB3 and leptin peptides on integrin αvβ3 gene expression, FAK activation and adhesion in thyroid cancer cell lines (**A**) Anaplastic, (**B**) papillary and (**C**) follicular thyroid cancer cells were treated with either leptin or OB3 for 24 h. Cells were harvested and total RNA was extracted. qPCR for *integrin αv* and β3 was conducted as described in the Materials and Methods. (**D**) Different thyroid cancer cells were incubated with 10 μM OB3, or 0.625 μM leptin for 30 min. Total cell extracts were prepared, proteins separated by PAGE, then immunoblotted with anti-phosphorylated FAK or total FAK antibody. β-Actin was used as an internal loading control. Immunoblots were visualized by enhanced chemiluminescence (Amersham Life Science, Arlington Heights, IL, USA) and digital imaging. (**E**) Thyroid cancer cells (5 × 10^5^/well) were starved in 0.1% serum-containing medium for 48 h. Cells were trypsinized and refed with medium with 0.625 μM leptin or 10 μM OB3 and cells were incubated at 37°C for 24 h. Cells were seeded into the 6-well cell culture plate for 6 h. The attachment cells were quantified using the fluorimetric detection system (Millipore). (Data were expressed as mean ± S.D. in triplicate. *p < 0.05, **p < 0.01, ***p < 0.001, were compared with control).

Integrin αvβ3 has been shown to be involved in cell migration and adhesion. We examine the effect of leptin on the expression of integrin. In anaplastic thyroid cancer cells, leptin reduced the expression of integrin β3 significantly and increased integrin αv significantly (Figure 5A). On the other hand, OB3 reduced the expression of integrin β3, but the increase of integrin αv was slight (Figure 5A) in the same cell culture. The effects of OB3 and leptin on the expression of integrin αvβ3 in papillary thyroid cancer cell lines were similar to those observed in anaplastic thyroid cancer cells (Figure 5B). Both OB3 and leptin reduced the expression of integrin β3 significantly in BHP18-21 and BHP2-7 cells (Figure 5B). Interestingly, OB3 did not affect integrin αvβ3 expression in follicular thyroid cancer FTC236 cells, but it increased β3 expression in FTC238 cells (Figure 5C). Leptin increased integrin β3 expression significantly only in FTC236 cells (Figure 5C); it decreased αv in FTC236 cells and both αv and β3 in FTC238 cells significantly (Figure 5C).

We also examined FAK activation downstream of integrin αvβ3. Interestingly, both leptin and OB3 reduced the activation of FAK in anaplastic thyroid cancer cells (Figure 5D). Studies of adhesion also confirmed that reduction of the expression of integrin β3 reduced the activation of FAK which consequently inhibited adhesion in anaplastic thyroid cancer cells (Figure 5E). On the other hand, both OB3 and leptin enhanced adhesive capability significantly in BHT18-21, FTC236 and FTC238 cells and slightly in FTC238 cells (Figure 5E). This indicates that neither OB3 nor leptin affected the expression of heterodimeric integrin αvβ3, an adhesion factor in papillary and follicular thyroid cancer cells, as noted above. These results suggest that the inhibition by leptin of expression of monomeric integrin β3 affected the FAK activation and thus reduced adhesion capability, presumably via another integrin heterodimer that contains β3.

### Leptin and OB3 affect differentially the circulating concentrations of pituitary trophic hormones in the intact mouse

Leptin has been shown by other investigators to increase circulating LH levels [33] and TSH levels [34], but not to affect FSH. We examined the effects of OB3 and leptin on the levels of LH, TSH and FSH in intact mice at 2 h and 48 h after leptin or OB3 administration. Results indicated that leptin (80 μg/kg) significantly, increased levels of FSH at 48 h and significantly decreased LH at 2 and 48 h after administration (Table 1). OB3 (1 mg/kg), however, did not significantly affect LH, but increased FSH (at 48 h) comparably to leptin. Leptin caused a small, but significant, increase in serum TSH levels at 48 h. OB3 administration did not increase circulating TSH, but was associated with a downward trend in TSH at 2 and 48 h.

**Table 1:** Leptin and OB3 affect the circulating levels of hormones

		Control	OB3 (1 mg/kg)	Leptin (1 μg/kg)
**FSH (ng/ml)**	0 hr	109.31 ± 3.07	82.71 ± 3.64	93.87 ± 5.69
	2 hrs	113.06 ± 3.07	87.3 ± 2.73	99.19 ± 19.4
	48 hrs	120.10 ± 1.93	116.44 ± 1.36**	147.56 ± 20.2***
**LH (ng/ml)**	0 hr	4.44 ± 0.45	4.48 ± 0.63	5.76 ± 0.18
	2 hrs	6.25 ± 0.12	6.1 ± 0.14	4.3 ± 0.29***
	48 hrs	4.36 ± 0.08	4.5 ± 0.12	4.17 ± 1.07**
**TSH (pg/ml)**	0 hr	113.52 ± 16.8	119.5 ± 30.9	118.4 ± 2.59
	2 hrs	105.3 ± 14.4	95.82 ± 39.8	119.9 ± 7.58
	48 hrs	115.1 ± 15.1	92.7 ± 6.27	128.7 ± 3.74*

(Mean ± SD, **p < 0.01, ***p < 0.001, were compared with 0 hr).

## DISCUSSION

We show here that the recently developed OB3 peptide did not induce cell proliferation and migration in various thyroid cancer cells. On the other hand, leptin enhanced cell migration and invasion in anaplastic thyroid cancer cells. Serum leptin has been studied in breast cancer patients and concentrations were higher in women with high-grade tumors [27]. Several studies indicate that leptin may be involved in carcinogenesis, cancer cell proliferation, cell migration and invasion [35–37]. An exception appears to be pancreatic cancer cells where leptin inhibits the growth of Mia-PaCa and PANC-1 pancreatic cancer cells through unknown mechanisms [29].

In contrast to the parent peptide hormone, OB3-leptin peptide did not stimulate cancer cell proliferation in MTT assay in HeLa cells (data not shown) or in all of the thyroid cancer cells examined by us (Figure 1A, 1B). These two peptides did not increase the expression of genes involved in proliferation (Figure 1C, 1D and 1E) in thyroid cancer cells.

Leptin not only stimulated expression of genes involved in invasion (Figure 3A), but also promoted invasion in the Transwell assay (Figure 3D) in anaplastic thyroid cancer cells. Both VEGF and FGF play important roles in migration and angiogenesis. Leptin has been shown to induce VEGF and FGF in different cancer cell types *in vitro*. High levels of leptin, VEGF and FGF exist concomitantly in clinical samples. Our results also showed that leptin induced VEGF expression (Figure 3A), whereas OB3 did not (Figure 3A). In the chick chorioallantoic membrane (CAM) system, however, OB3 and leptin both stimulated angiogenesis, and both inhibited FGF-induced angiogenesis in CAM assay (P. Grasso, unpublished observation).

Like leptin, OB3 peptide induced phosphorylation (activation) of ERK1/2 and PI-3K and phosphorylation of Ser-727 and Tyr-705 of STAT3 in HeLa cells [10]. However OB3 did not induce activation of ERK1/2, PI3K and STAT3 in anaplastic thyroid cancer and papillary thyroid cancer cells (results not shown). Unlike OB3, leptin induced activation of STAT3 via phosphorylation of ERK1/2 and of Tyr705 of STAT3 (Figure 4A). Indeed, pharmacologic inhibition of ERK1/2 and STAT3, but not PI3K, signaling inhibited leptin-induced invasion in anaplastic thyroid cancer cells (Figure 4B). These results suggest that leptin activates ERK1/2 to phosphorylate Tyr-705 in STAT3; this is essential to triggering of leptin-induced *MMP2*, *MMP9* and *VEGF* expression and consequent cell invasion. Hormones and growth factors activate ERK1/2 that supports cancer cell proliferation and metastasis. Thyroid hormone induces cancer cell growth in breast [28, 38], thyroid [28, 39], and glioblastoma [28, 40] via activated ERK1/2. Estrogen [41] and DHT [42] activate ERK1/2 and consequent cell proliferation in breast cancer cells. In addition, angiogenesis which plays an important role in cancer cell metastasis induced by thyroid hormone is activated ERK1/2-dependent. Aberrant activation of STAT3 has been reported to promote cancer progression in many human cancers [16]. Obesity-induced thyroid tumor growth and cancer progression have been shown to be mediated by the enhancement of phosphorylation of oncogenic JAK2 and STAT3 transcription factors [16, 32]. Recent evidence also suggests that inhibition of the STAT3 activity may be a treatment strategy for obesity-induced thyroid cancer [43]. Thyroid hormone stimulates STAT3 phosphorylation and potentiates EGF-induced STAT3 phosphorylation in HeLa cells [44]. Hypothyroid mice have increased expression of leptin receptor Ob-R and decreased suppressor of cytokine signaling 3 transcript levels. STAT3 activation is also reduced in such animals with leptin treatment [45].

PI3K has also been shown to be involved in leptin-induced cancer proliferation. Insulin stimulates leptin release through the PI3K/Akt pathway, an effect that is Ca^2+^-requiring [46]. Leptin-induced increase in hepatic sympathetic outflow also depends on PI3K [47]. The PI3K/Akt pathway also mediates leptin-induced neuroprotection [48].

Clinical studies have shown that there is a strong correlation of the leptin expression with the Ob-R expression in thyroid cancer cells. Leptin and Ob-R have negative prognostic significance in papillary thyroid cancer, while Ob-R may play a protective role in anaplastic thyroid cancer [30]. Our results demonstrate that leptin stimulates invasiveness and reduced adhesion of anaplastic thyroid cancer cells (Figures 3E and 5D). Although leptin and Ob-R expression are strongly correlated with older age, larger tumor size, nodal metastasis and advanced stage in clinical studies of papillary thyroid cancer, our results and those of others show that leptin does not stimulate cell proliferation (Figure 1; [26]). In follicular thyroid cancer patients, expression of leptin or Ob-R expression are not correlated with recurrence or metastasis during the follow-up [30]. This is consistent with our own experimental observations that leptin did not stimulate proliferation in follicular thyroid cancer cells (Figure 1).

The effects of leptin and OB3 on carbohydrate metabolism-related gene expression were variable in the thyroid cancer cell lines studied (Figure 2). Although GLUT1expression is related to breast cancer invasion, OB3-induced GLUT1 expression (Figure 2C) was not related to proliferation and invasion of follicular thyroid cancer cells.

Work by others in pituitary cells has shown that leptin increases TSH secretion (34) and we confirmed this observation in intact mice (Table 1). OB3 lacks this effect. In fact, there was a nonsignificant downward trend in circulating TSH in mice treated with OB3. This issue is of relevance to differentiated thyroid cancer, where TSH is a trophic factor (28).

In summary, leptin in anaplastic thyroid cancer cells not only increased expression of MMP2, MMP9 and VEGF in the current studies, supporting angiogenesis, but also induced cancer cell invasiveness and reduced adhesion. On the other hand, OB3 lacked effects on carcinogenesis in the thyroid cancer cell systems studied. Although OB3 may have angiogenic potential, it inhibited FGF-induced angiogenesis. Our observations need to be reproduced in other cancer cell systems, but the present study provides additional assurance of safety of OB3 over leptin as a potential anti-obesity pharmaceutical. Further, OB3 administration should reduce endogeous leptin production, reducing the likelihood of trophic contributions of host leptin to existing tumors.

## MATERIALS AND METHODS

### OB3 and leptin peptide

Leptin peptide was purchased from Sigma. OB3 peptide was synthesized on a Rainin model PS3 automated peptide synthesizer (Ridgefield, NJ, USA) by the solid phase method. Fluorenylmethoxycarbonyl-protected l- or d-amino acids (Rainin, Ridgefield, NJ, USA) were used. The peptides were assembled on Rink's 4,2′,4′-dimethyloxyphenol fluorenylmethoxycarbonyl-aminomethyl-phenoxy-amide resin (Fisher Scientific, Springfield, NJ). Completed peptide amides were cleaved from the resin with trifluoroacetic acid (84%), using sterile deionized water (4%), ethanedithiol (4%), anisole (4%), and thioanisole (4%) as scavengers. The cleaved peptides were precipitated with anhydrous ether and dried by lyophilization. The peptide amides were purified to 98% on a Rainin Dynamax preparative column (21.4 mm × 325 cm; C18; 300-A pore diameter). The final peptide products were evaluated for purity by reverse phase liquid chromatography on a Rainin Dynamax analytical column (4.6 mm × 325 cm; C18; 300-A pore diameter) using a linear acetonitrile gradient (0–100%) containing 0.05% trifluoroacetic acid and a flow rate of 1 mL/min. Each peptideamide was represented as a single peak in the chromatographic profile. Fidelity of synthesis was confirmed by mass spectralanalysis.

### Cell lines

Human thyroid papillary cancer cell lines BHP2-7 and BHP18-21 were generously provided by Dr. Jerome M. Hershman (West Los Angeles VA Medical Center, CA, USA). Human thyroid follicular cancer cell lines FTC236 and FTC238 were kindly provided by Dr. Orlo Clark (University of California at San Francisco-Mt. Zion Medical Center, San Francisco, CA, USA). The human anaplastic thyroid cancer cell line, ARO, was generously provided by Dr. Oscar K. Lee (Stem cell Research Center, National Yang-Ming University, Taipei, Taiwan). Cell lines were maintained for study in RPMI 1640 (for ARO, BHP2-7 and BHP18-21), or 50% DMEM/50% Ham's F-12 plus 10 mU/mL of TSH (Sigma-Aldrich, St. Louis, MO, USA) (for FTC236 and FTC238) in a 5% CO_2_/95% O_2_ incubator at 37°C. Prior to treatment, cells were placed in 0.25% hormone-stripped FBS-containing medium for 2 days.

### Cell viability assay

Cells (5 × 10^3^ cells per well) were seeded in 96-well plates and treated or untreated (controls) for 72 h. Cell proliferation was determined by incubating the cells with 200 mL of fresh medium containing 1 mg/mL 3-(4,5-dimethylthiazol-2-yl)-2,5-diphenyltetrazolium bromide (MTT) (Sigma-Aldrich) for 4 h at 37°C. After removal of the MTT solution, the resulting formazan crystals were dissolved completely in an ethanol/dimethyl sulfoxide mixture (1:1) and the plates were read using a microplate reader (Anthos 2010; Biochrom, Cambridge, UK) by measuring the absorbance at 490 nm. Triplicate wells were assayed for each experiment and three independent experiments were performed. Data are expressed as the mean of OD490 ± SD.

### Western blotting

To examine the effects of leptin and OB3-leptin peptide in these thyroid cancer cell lines, we performed western blot analysis to quantify the protein expression levels of pERK1/2, ERK2, pSTAT3(y705), pSTAT3(s727), pPI3K(p85), pFAK(y397), and FAK in the total cell lysates. Protein samples were resolved on a 10% sodium dodecyl sulfate polyacrylamide gel (SDS-PAGE). Twenty μg of protein was loaded in each well with 5× sample buffer, and the protein samples were resolved by electrophoresis at 100 V for 2 h. The resolved proteins were transferred from the polyacrylamide gel to Millipore Immobilon-PSQ Transfer PVDF membranes (Millipore, Billerica, MA, USA) with the Mini Trans-Blot^®^ Cell (Bio-Rad Laboratories, Inc., Hercules, CA, USA). The membranes were blocked with a solution of 2% fetal bovine serum in Tris-buffered saline. The membranes were incubated with primary antibodies to phospho-p44/42 MAPK (pERK1/2), ERK2, pSTAT3(y705), pSTAT3(s727), pPI3K(p85), pFAK(y397) (Cell Signaling Technology, Inc., Beverly, MA, USA), FAK, GAPDH (GeneTex International Corporation, Hsinchu City, Taiwan), at 4°C overnight and washed, and the proteins were detected with HRP-conjugated secondary antibodies and ImmobilonTM Western HRP Substrate Luminol Reagent (Millipore). Images of the western blots were visualized and recorded by BioSpectrum^®^ Imaging System (UVP, LLC, Upland, CA, USA).

### Quantitative real-time PCR

To examine mRNA expression, we treated leptin or OB3-leptin peptide in these thyroid cancer cell lines for 24 h. Total RNA was extracted and genomic DNA was also eliminated with Illustra RNAspin Mini RNA Isolation Kit (GE Healthcare Life Sciences, Buckinghamshire, United Kingdom). One μg of DNase I-treated total RNA was reverse-transcribed with RevertAid H Minus First Strand cDNA Synthesis Kit (Life Technologies Corporation, Carlsbad, California, USA) into cDNA, and used as the template for real-time PCR reactions and analysis. The real time PCR reactions were performed using QuantiNova^™^ SYBR^®^ Green PCR Kit (QIAGEN) on CFX Connect™ Real-Time PCR Detection System (Bio-Rad Laboratories, Inc., Hercules, CA, USA). This involved an initial denaturation at 95°C for 5 min, followed by 40 cycles of denaturing at 95°C for 5 sec and combined annealing/extension at 60°C for 10 sec, as described in the manufacturer's instructions. The primer sequences were shown as following: *Homo sapiens* proliferating cell nuclear antigen (*PCNA*), forward 5′-TCTGAGGGCTTCGACACCTA-3′ and reverse 5′-TCATTGCCGGCGCATTTTAG-3′ (Accession No.: BC062439.1); *Homo sapiens* v-myc avian myelocytomatosis viral oncogene homolog, (*c-Myc*), forward 5′-TTCGGGTAGTGGAAAACCAG-3′ and reverse 5′-CAGCAGCTCGAATTTCTTCC-3′ (Accession No.: NM_002467); *Homo sapiens* myeloid cell leukemia 1 (*MCL1*), forward 5′-CCAAGAAAGCTGCATCGAACC-3′ and reverse 5′-CAAACCCATCCCAGCCTCTT-3′ (Accession No.: NM_021960.4); *Homo sapiens* solute carrier family 2 (facilitated glucose transporter), member 1, (Glut1), forward 5′-ATGGGCTTCTCGAAACTGGG-3′ and reverse 5′-CCGCAGTACACACCGATGAT-3′ (Accession No.: NM_006516.2); *Homo sapiens* solute carrier family 2 (facilitated glucose transporter), member 2, (Glut2), forward 5′-TGAAGCCACAGGTTGCTGAG-3′ and reverse 5′-GGCTACCCAGACCTGAGAGT-3′ (Accession No.: NM_000340.1); *Homo sapiens* solute carrier family 2 (facilitated glucose transporter), member 5, (Glut5), forward 5′-GGGGCACCCACTTACTTAGC-3′ and reverse 5′-GGCCATGCCAAATAACAGCC-3′ (Accession No.: NM_003039.2); *Homo sapiens* hexokinase 1 (HK1), forward 5′-CGCAGCTCCTGGCCTATTAC-3′ and reverse 5′-GAGCCGCATGGCATAGAGAT-3′ (Accession No.: NM_000188.2); *Homo sapiens* matrix metallopeptidase 2 (*MMP2*), forward 5′-ATCCAGACTTCCTCAGGCGG-3′ and reverse 5′-CCTGGCAATCCCTTTGTATGTT-3′ (Accession No.: NM_004530.5); *Homo sapiens* matrix metallopeptidase 9 (*MMP9*), forward 5′-TGTACCGCTATGGTTACACTCG-3′ and reverse 5′-GGCAGGGACAGTTGCTTCT-3′ (Accession No.: NM_004994.2); *Homo sapiens* vascular endothelial growth factor A (*VEGF-A*), forward 5′-TACCTCCACCATGCCAAGTG-3′ and reverse 5′-GATGATTCTGCCCTCCTCCTT-3′ (Accession No.: NM_001204384.1); *Homo sapiens* integrin, alpha v (*Integrin αv*), forward 5′-TCCGATTCCAAACTGGGAGC-3′ and reverse 5′-AAGGCCACTGAAGATGGAGC-3′ (Accession No.: NM_002210.4); *Homo sapiens* integrin, beta 3 (*Integrin β3*), forward 5′-CTGGTGTTTACCACTGATGCCAAG-3′ and reverse 5′-TGTTGAGGCAGGTGGCATTGAAGG-3′ (Accession No.: NM_000212.2); *Homo sapiens* glyceraldehyde-3-phosphate dehydrogenase (*GAPDH*), forward 5′-TGCCAAATATGATGACATCAAGAA-3′ and reverse 5′-GGAGTGGGTGTCGCTGTTG-3′ (Accession No.NM_002046). Calculations of relative gene expression (normalized to GAPDH reference gene) were performed according to the ΔΔCT method. Fidelity of the PCR reaction was determined by melting temperature analysis.

### Transwell cell migration and invasion assay

Cell migration and invasion assay were conducted in the Transwell system (Corning Incorporated, Corning, NY, USA). Briefly, cells were trypsinized and adjusted to 8 × 10^5^ cells/ml of cell suspension. Cells in a 200-μl volume were seeded into the upper chamber of Transwell, and added 800 μl medium with 10% stripped FBS in the lower chamber. Cells were then cultured at 37°C for 48 h and the cells on the surface of the up chamber were swabbed with a cotton swab and the cells under the surface of the low chamber were stained with crystal violet (0.1%). Cells were then photographed in an inverted microscope for capturing pictures and counted for migrating cell numbers. In addition, 50 μl BD Matrigel^™^ (BD Biosciences, San Jose, CA, USA) was added onto each upper chamber of Transwells and the Transwells were placed in a 37°C incubator for 2–3 h to solidify the Matrigel. The tumor cell invasion capacity was then assessed similar to the migration assay.

### Animal study

Nude mice (BALB/cAnN.Cg-Foxn1^nu^/CrlNarl) were purchased form National Laboratory Animal Center (Taipei, Taiwan) and were housed in a reserved, pathogen-free facility and were handled in accordance with the protocols approved by the Institutional Animal Care and Use Committee of National Defense Medical Center, Taipei, Taiwan (IACUC-15-340). To determine whether leptin or OB3 affect circulating levels of TSH, LH or FSH, we injected mice intraperitoneally with 80 μg/kg leptin or 1 mg/kg OB3. Ten-week-old male nude mice (*n* = 15), weighing between 20 and 25 g, were randomly divided into three treatment groups, defined as follows: control group (saline injection, *n* = 5), leptin group (80 μg/kg leptin injection, *n* = 5) and OB3 group (1 mg/kg OB3, *n* = 5). Blood samples were collected on 7 d before injection and 2 h and 48 h after injection. Serum samples were separated by centrifugation and stored at −80°C.

### Detection of TSH, FSH and LH by competition ELISA

Levels of TSH, FSH and LH in aliquots of serum were measured by competition ELISA with commercial test kits: Thyroid Stimulating Hormone (TSH) ELISA Kit (Catalog No.: ABIN415519, Antibodies-online Inc., Atlanta, GA, USA); Mouse FSH ELISA Kit (Catalog No.: MBS2507988, MyBioSource Inc., San Diego, CA, USA); LH ELISA Kit (Catalog No.: ABIN415551, Antibodies-online Inc.). All ELISA examinations were carried out according to the manufacturer's instructions.

### Data analysis and statistics

Western blotting densities were measured and gene expression of quantitative real-time PCR were analyzed by IBM^®^ SPSS^®^ Statistics software version 19.0 (SPSS Inc., Chicago, IL, USA). Student's *t*-test was conducted and considered significant at *p*-values < 0.05 (*), 0.005 (**) and 0.001 (***).

## References

[1] Padidar S, Farquharson AJ, Williams LM, Kearney R, Arthur JR, Drew JE (2012). High-fat diet alters gene expression in the liver and colon: links to increased development of aberrant crypt foci. Dig Dis Sci.

[2] Basen-Engquist K, Chang M (2011). Obesity and cancer risk: recent review and evidence. Curr Oncol Rep.

[3] Trevellin E, Scarpa M, Carraro A, Lunardi F, Kotsafti A, Porzionato A, Saadeh L, Cagol M, Alfieri R, Tedeschi U, Calabrese F, Castoro C, Vettor R (2015). Esophageal adenocarcinoma and obesity: peritumoral adipose tissue plays a role in lymph node invasion. Oncotarget.

[4] Garofalo C, Surmacz E (2006). Leptin and cancer. J Cell Physiol.

[5] Vona-Davis L, Rose DP (2007). Adipokines as endocrine, paracrine, and autocrine factors in breast cancer risk and progression. Endocr Relat Cancer.

[6] Chen C, Chang YC, Liu CL, Chang KJ, Guo IC (2006). Leptin-induced growth of human ZR-75-1 breast cancer cells is associated with up-regulation of cyclin D1 and c-Myc and down-regulation of tumor suppressor p53 and p21WAF1/CIP1. Breast Cancer Res Treat.

[7] Chen C, Chang YC, Liu CL, Liu TP, Chang KJ, Guo IC (2007). Leptin induces proliferation and anti-apoptosis in human hepatocarcinoma cells by up-regulating cyclin D1 and down-regulating Bax via a Janus kinase 2-linked pathway. Endocr Relat Cancer.

[8] Huang CY, Yu HS, Lai TY, Yeh YL, Su CC, Hsu HH, Tsai FJ, Tsai CH, Wu HC, Tang CH (2011). Leptin increases motility and integrin up-regulation in human prostate cancer cells. J Cell Physiol.

[9] Nepal S, Kim MJ, Hong JT, Kim SH, Sohn DH, Lee SH, Song K, Choi DY, Lee ES, Park PH (2015). Autophagy induction by leptin contributes to suppression of apoptosis in cancer cells and xenograft model: involvement of p53/FoxO3A axis. Oncotarget.

[10] Lin HY, Yang SH, Tang HY, Cheng GY, Davis PJ, Grasso P (2014). Biologically active leptin-related synthetic peptides activate STAT3 via phosphorylation of ERK1/2 and PI-3K. Peptides.

[11] Rozhavskaya-Arena M, Lee DW, Leinung MC, Grasso P (2000). Design of a synthetic leptin agonist: effects on energy balance, glucose homeostasis, and thermoregulation. Endocrinology.

[12] Waldrop MA, Leinung MC, Lee DW, Grasso P (2010). Intranasal delivery of mouse [D-Leu-4]-OB3, a synthetic peptide amide with leptin-like activity, improves energy balance, glycaemic control, insulin sensitivity and bone formation in leptin-resistant C57BLK/6-m db/db mice. Diabetes Obes Metab.

[13] Novakovic ZM, Leinung MC, Lee DW, Grasso P (2010). Oral delivery of mouse [d-Leu-4]-OB3, a synthetic peptide amide with leptin-like activity, in male C57BL/6J wild-type and ob/ob mice: effects on energy balance, glycaemic control and serum osteocalcin levels. Diabetes Obes Metab.

[14] Feldt-Rasmussen U (2007). Thyroid and leptin. Thyroid.

[15] Cheng SP, Liu CL, Hsu YC, Chang YC, Huang SY, Lee JJ (2012). Regulation of leptin receptor expression in human papillary thyroid cancer cells. Biomed Pharmacother.

[16] Cheng SP, Yin PH, Hsu YC, Chang YC, Huang SY, Lee JJ, Chi CW (2011). Leptin enhances migration of human papillary thyroid cancer cells through the PI3K/AKT and MEK/ERK signaling pathways. Oncol Rep.

[17] Kim WG, Park JW, Willingham MC, Cheng SY (2013). Diet-induced obesity increases tumor growth and promotes anaplastic change in thyroid cancer in a mouse model. Endocrinology.

[18] Yu H, Pardoll D, Jove R (2009). STATs in cancer inflammation and immunity: a leading role for STAT3. Nat Rev Cancer.

[19] Zhang X, Yue P, Page BD, Li T, Zhao W, Namanja AT, Paladino D, Zhao J, Chen Y, Gunning PT, Turkson J (2012). Orally bioavailable small-molecule inhibitor of transcription factor Stat3 regresses human breast and lung cancer xenografts. Proc Natl Acad Sci U S A.

[20] Weiner TM, Liu ET, Craven RJ, Cance WG (1993). Expression of focal adhesion kinase gene and invasive cancer. Lancet.

[21] Owens LV, Xu L, Craven RJ, Dent GA, Weiner TM, Kornberg L, Liu ET, Cance WG (1995). Overexpression of the focal adhesion kinase (p125FAK) in invasive human tumors. Cancer Res.

[22] Brooks PC, Clark RA, Cheresh DA (1994). Requirement of vascular integrin alpha v beta 3 for angiogenesis. Science.

[23] Brooks PC, Stromblad S, Klemke R, Visscher D, Sarkar FH, Cheresh DA (1995). Antiintegrin alpha v beta 3 blocks human breast cancer growth and angiogenesis in human skin. J Clin Invest.

[24] Chen HC, Appeddu PA, Isoda H, Guan JL (1996). Phosphorylation of tyrosine 397 in focal adhesion kinase is required for binding phosphatidylinositol 3-kinase. J Biol Chem.

[25] Ratke J, Entschladen F, Niggemann B, Zanker KS, Lang K (2010). Leptin stimulates the migration of colon carcinoma cells by multiple signaling pathways. Endocr Relat Cancer.

[26] Cheng SP, Yin PH, Chang YC, Lee CH, Huang SY, Chi CW (2010). Differential roles of leptin in regulating cell migration in thyroid cancer cells. Oncol Rep.

[27] Akinci M, Kosova F, Cetin B, Aslan S, Ari Z, Cetin A (2009). Leptin levels in thyroid cancer. Asian J Surg.

[28] Lin HY, Chin YT, Yang YCSH, Lai HY, Whang-Peng J, Liu LF, Tang HY, Davis PJ (2016). Thyroid hormone, cancer, and apoptosis. Comprehensive Physiology.

[29] Gainsford T, Willson TA, Metcalf D, Handman E, McFarlane C, Ng A, Nicola NA, Alexander WS, Hilton DJ (1996). Leptin can induce proliferation, differentiation, and functional activation of hemopoietic cells. Proc Natl Acad Sci U S A.

[30] Barrichon M, Hadi T, Wendremaire M, Ptasinski C, Seigneuric R, Marcion G, Delignette M, Marchet J, Dumas M, Sagot P, Bardou M, Garrido C, Lirussi F (2015). Dose-dependent biphasic leptin-induced proliferation is caused by non-specific IL-6/NF-kappaB pathway activation in human myometrial cells. Br J Pharmacol.

[31] Rossetti L, Massillon D, Barzilai N, Vuguin P, Chen W, Hawkins M, Wu J, Wang J (1997). Short term effects of leptin on hepatic gluconeogenesis and *in vivo* insulin action. J Biol Chem.

[32] Hill JW, Williams KW, Ye C, Luo J, Balthasar N, Coppari R, Cowley MA, Cantley LC, Lowell BB, Elmquist JK (2008). Acute effects of leptin require PI3K signaling in hypothalamic proopiomelanocortin neurons in mice. J Clin Invest.

[33] Dagklis T, Kouvelas D, Kallaras K, Papazisis G, Petousis S, Margioula-Siarkou C, Skepastianos P, Tarlatzis BC (2015). Leptin increases luteinizing hormone secretion of fasting female rats. Clin Exp Obstet Gynecol.

[34] Radwanska P, Kosior-Korzecka U (2014). Effect of leptin on thyroid-stimulating hormone secretion and nitric oxide release from pituitary cells of ewe lambs *in vitro*. J Physiol Pharmacol.

[35] Kato S, Abarzua-Catalan L, Trigo C, Delpiano A, Sanhueza C, Garcia K, Ibanez C, Hormazabal K, Diaz D, Branes J, Castellon E, Bravo E, Owen G (2015). Leptin stimulates migration and invasion and maintains cancer stem-like properties in ovarian cancer cells: an explanation for poor outcomes in obese women. Oncotarget.

[36] Fan Y, Gan Y, Shen Y, Cai X, Song Y, Zhao F, Yao M, Gu J, Tu H (2015). Leptin signaling enhances cell invasion and promotes the metastasis of human pancreatic cancer via increasing MMP-13 production. Oncotarget.

[37] Avtanski DB, Nagalingam A, Kuppusamy P, Bonner MY, Arbiser JL, Saxena NK, Sharma D (2015). Honokiol abrogates leptin-induced tumor progression by inhibiting Wnt1-MTA1-beta-catenin signaling axis in a microRNA-34a dependent manner. Oncotarget.

[38] Tang HY, Lin HY, Zhang S, Davis FB, Davis PJ (2004). Thyroid hormone causes mitogen-activated protein kinase-dependent phosphorylation of the nuclear estrogen receptor. Endocrinology.

[39] Lin HY, Tang HY, Shih A, Keating T, Cao G, Davis PJ, Davis FB (2007). Thyroid hormone is a MAPK-dependent growth factor for thyroid cancer cells and is anti-apoptotic. Steroids.

[40] Lin HY, Sun M, Tang HY, Lin C, Luidens MK, Mousa SA, Incerpi S, Drusano GL, Davis FB, Davis PJ (2009). L-Thyroxine vs. 3,5,3′-triiodo-L-thyronine and cell proliferation: activation of mitogen-activated protein kinase and phosphatidylinositol 3-kinase. Am J Physiol Cell Physiol.

[41] Zhang S, Cao HJ, Davis FB, Tang HY, Davis PJ, Lin HY (2004). Oestrogen inhibits resveratrol-induced post-translational modification of p53 and apoptosis in breast cancer cells. Br J Cancer.

[42] Chin YT, Yang SH, Chang TC, Changou CA, Lai HY, Fu E, HuangFu WC, Davis PJ, Lin HY, Liu LF (2015). Mechanisms of dihydrotestosterone action on resveratrol-induced anti-proliferation in breast cancer cells with different ERalpha status. Oncotarget.

[43] Park JW, Han CR, Zhao L, Willingham MC, Cheng SY (2016). Inhibition of STAT3 activity delays obesity-induced thyroid carcinogenesis in a mouse model. Endocr Relat Cancer.

[44] Lin HY, Shih A, Davis FB, Davis PJ (1999). Thyroid hormone promotes the phosphorylation of STAT3 and potentiates the action of epidermal growth factor in cultured cells. Biochem J.

[45] Groba C, Mayerl S, van Mullem AA, Visser TJ, Darras VM, Habenicht AJ, Heuer H (2013). Hypothyroidism compromises hypothalamic leptin signaling in mice. Mol Endocrinol.

[46] Wang Y, Ali Y, Lim CY, Hong W, Pang ZP, Han W (2014). Insulin-stimulated leptin secretion requires calcium and PI3K/Akt activation. Biochem J.

[47] Tanida M, Yamamoto N, Morgan DA, Kurata Y, Shibamoto T, Rahmouni K (2015). Leptin receptor signaling in the hypothalamus regulates hepatic autonomic nerve activity via phosphatidylinositol 3-kinase and AMP-activated protein kinase. J Neurosci.

[48] Zhang J, Deng Z, Liao J, Song C, Liang C, Xue H, Wang L, Zhang K, Yan G (2013). Leptin attenuates cerebral ischemia injury through the promotion of energy metabolism via the PI3K/Akt pathway. J Cereb Blood Flow Metab.

