# Correction: Novel leptin OB3 peptide-induced signaling and progression in thyroid cancers: Comparison with leptin

**DOI:** 10.18632/oncotarget.18067

**Published:** 2017-05-22

**Authors:** Yu-Chen SH Yang, Yu-Tang Chin, Meng-Ti Hsieh, Hsuan-Yu Lai, Chien-Chih Ke, Dana R. Crawford, Oscar K. Lee, Earl Fu, Shaker A. Mousa, Patricia Grasso, Leroy F. Liu, Heng-Yu Chang, Heng-Yuan Tang, Hung-Yun Lin, Paul J. Davis

**Present:** A typographical error was made on Table 1 regarding the concentration of leptin injected to mice.

**Correct:** The proper Table is given below.

Original article: Oncotarget. 2016; 7:27641-27654. doi: 10.18632/oncotarget.8505

**Table 1:** Leptin and OB3 affect the circulating levels of hormones

		Control	OB3 (1 mg/kg)	Leptin (80 μg/kg)
FSH (ng/ml)	0 hr	109.31 ± 3.07	82.71 ± 3.64	93.87 ± 5.69
	2 hrs	113.06 ± 3.07	87.3 ± 2.73	99.19 ± 19.4
	48 hrs	120.10 ± 1.93	116.44 ± 1.36**	147.56 ± 20.2***
LH (ng/ml)	0 hr	4.44 ± 0.45	4.48 ± 0.63	5.76 ± 0.18
	2 hrs	6.25 ± 0.12	6.1 ± 0.14	4.3 ± 0.29***
	48 hrs	4.36 ± 0.08	4.5 ± 0.12	4.17 ± 1.07**
TSH (pg/ml)	0 hr	113.52 ± 16.8	119.5 ± 30.9	118.4 ± 2.59
	2 hrs	105.3 ± 14.4	95.82 ± 39.8	119.9 ± 7.58
	48 hrs	115.1 ± 15.1	92.7 ± 6.27	128.7 ± 3.74*

(Mean ± SD, ** *p* < 0.01, *** *p* < 0.001, were compared with 0 hr)

